# Autopsy Findings in Conjoined Twin with Single Heart and Single Liver

**DOI:** 10.1155/2012/129323

**Published:** 2012-08-26

**Authors:** Kar Asaranti, Mohanty Pranati, Kar Tushar, Behera Jagadish, Behera Susmita, Nayak Amarendra

**Affiliations:** ^1^Department of Pathology, S.C.B. Medical College, Cuttack, Odisha 753007, India; ^2^Department of O&G, S.C.B. Medical College, Cuttack, Odisha 753007, India; ^3^Department of F.M.T., S.C.B. Medical College, Cuttack, Odisha 753007, India

## Abstract

Thoracoomphalopagus is the commonest type of conjoined twin where the bodies are fused from upper chest to lower chest. The autopsy done can help counsil the parents for further pregnancies and determine the prognosis depending upon the type of cardiac anomaly by Seo classification when detected antenatally. We describe the detail pathological autopsy of such a case with single heart and single liver. A detail autopsy was done on the twin fetus. The twins shared a single heart and sometimes the liver and part of digestive system. The combined weight was 4.1 KG. Both were full-term male babies joined from below the nipple till umbilicus. Autopsy in conjoined twins helps in deciding the type of fusion of the body and also of the heart and great vessels. It can help in counseling parents about future pregnancies that there is no chance of recurrence of this abnormality and no need to be scared.

## 1. Introduction

In conjoined twin, a rare anomaly refers to an incomplete splitting of monozygotic twins after 12 days of embryogenesis. It is seen in 10.25 per million births. 75% of them occur in females [1]. They are classified according to the point of union; the label used is the Greek word pagus, which means “that which is fixed.” The most common varieties encountered were thoracoomphalopagus (28%), thoracopagus (18.5%), omphalopagus (10%), parasitic twins (10%) and craniopagus (6%) [2]. Thoracoomphalopagus is a variety of conjoined twins where two bodies are fused from upper chest to the lower chest. They usually share a heart and sometimes the liver and part of digestive system. The condition is more frequently found among females, with a ratio of 3 : 1. We present the autopsy findings in a case of thoracoomphalopagus who were males.

## 2. Case History

**Figure 1:**
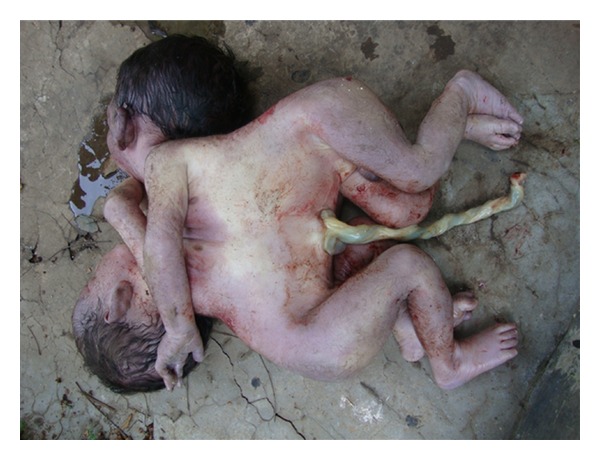
Photograph of babies joined at thorax.

**Figure 2:**
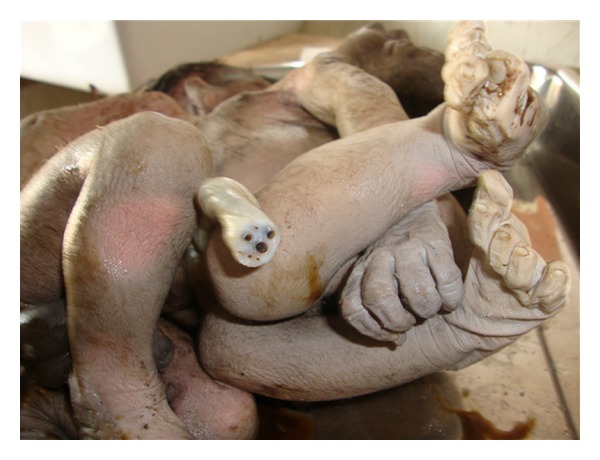
Photograph of umbilical cord.

A 32-year-old multigravida, P3L2 with gestational age of 35 weeks presented in labour with pain abdomen for 6 hours and show bloody vaginal discharge. She did not have any antenatal checkups and she could not appreciate fetal movements for last two days. After admission to labour room, pervaginum-examination was done. Cervix was fully effaced, fully dilated, and head was palpated. After half an hour she delivered the twin vaginally. Both were still born. A detail pathological autopsy was done. The combined weight was 4.1 KG. Both were full-term male babies joined from below the nipple till umbilicus (Figure 1). Placenta was single weighing 750 gms. One umbilical cord was shared by both. It had one artery and four veins (Figure 2). Nipples were separate, meconium was present, and signs of maceration were seen. Nails were up to tip of fingers, lanugo hair present in back, thighs, and arms. Anus was open, lips and palate was normal, no webbing of neck, palmar creases were normal, and fontanelle were present.

**Figure 3:**
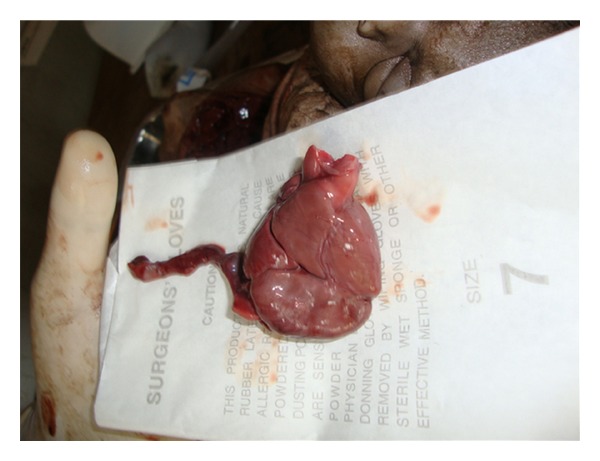
Photograph of single enlarged heart.

**Figure 4:**
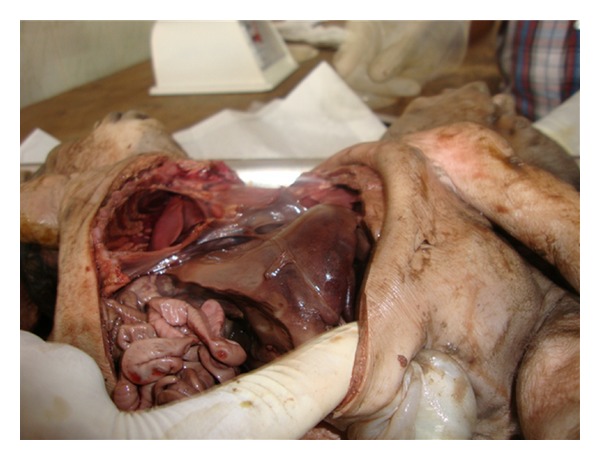
Abdominal cavity showing single liver.

 Fetus-1 (tied with a piece of gauge to distinguish from fetus 2)-crown-rump length was 28 cms, crown-heel length-44 cms, head circumference 33 cms, midarm circumference 8 cms. Fetus-2-crown-heel length was 45 cms and head circumference 38 cms. Other measurements were as fetus 1. Combined chest circumference was 38 cms. After skin incision and reflection, 2 sternums were seen. Two pleural cavities with 2 lungs in each were seen. Single heart was found weighing 150 gms and measuring 5 cms in length (Figure 3). One peritoneal cavity was seen containing two intestines. but one enlarged liver was present (Figure 4). Two gallbladders were present, separately on undersurface of liver. Tissue's from myocardium and liver were taken for histopathological examination. No abnormality could be found after light microscopic examination of hematoxylin and eosin stained sections.

## 3. Discussion

The earliest record of conjoined twin is that of Biddenden maids born in 1100CE, in England, and the most famous ones were the Siamese twins “Chang and Eng Bunker” born in 1811 in Siam who were xiphopagus, joined at lower chest by a narrow band of flesh which connected their livers. Their separation was not possible then though in modern days it would have been done easily.

 After the blastula stage, between 13th and 15th days after fertilization, the complete separation of inner cell mass within the chorionic mass does not occur, and nonseparated parts of otherwise normal twins remain fused throughout the development [3]. 39% of conjoined twins are still born, and 34% die within first day of life. In thoraco-omphalopagus, the degree of fusion of the heart determines the prognosis. Seo et al. introduced a new classification of CVS in conjoined twins. It has 5 types and is based on the degree of fusion and symmetry of heart and great vessels [4]. Since in the present case, there was fusion of atria and ventricles resulting in total two atria and two ventricles in single heart, it fits to type IV of Seo classification.

 Autopsy in conjoined twins helps in deciding the type of fusion of the body and also of the heart and great vessels. The prognosis is worst in thoracoomphalopagus especially with a single heart of Seo classification (Type IV and V). This can help in determining the chances of survival in future cases where the diagnosis can be done antenatally by radiology.

 Method of delivery depends upon prenatal assessment. Cesarean section is recommended in most third trimester deliveries. Vaginal delivery is reserved for still births and for forms of conjoined twins that are incompatible with life. The present case was an unbooked case and came at term and delivered vaginally. The separation of living conjoined twins is rarely successful. But, the perioperative mortality rate for thoracoomphalopagus has decreased from 58.31% between 1975 and 1979 to 26% in 1980 and 1987 [3]. Prognosis is better if separation is delayed till the infants are 6–12 months of age. Since the etiology of conjoined twins is sporadic incomplete separation, there is no increased risk in subsequent pregnancies, and the parents of conjoined twins were counseled accordingly.
